# Quantitation of normal metabolite concentrations in six brain regions by *in-vivo* ^1^H-MR spectroscopy

**DOI:** 10.4103/0971-6203.62128

**Published:** 2010

**Authors:** Ludovico Minati, Domenico Aquino, Maria Grazia Bruzzone, Alessandra Erbetta

**Affiliations:** 1 Scientific Department Fondazione IRCCS Istituto Neurologico Carlo Besta, Milano, Italy; 2 Neuroradiology Unit, Fondazione IRCCS Istituto Neurologico Carlo Besta, Milano, Italy

**Keywords:** Brain metabolites, magnetic resonance spectroscopy, normal concentrations, quantitation

## Abstract

This study examined the concentrations of brain metabolites visible to *in-vivo* ^1^H-Magnetic Resonance Spectroscopy (^1^H-MRS) at 1.5 T in a sample of 28 normal subjects. Quantitation was attempted for inositol compounds, choline units, total creatine and N-acetyl moieties, using open-source software. Six brain regions were considered: frontal and parietal white matter, medial temporal lobe, thalamus, pons and cerebellum. Absolute concentrations were derived using tissue water as an internal reference and using an external reference; metabolite signal intensity ratios with respect to creatine were also calculated. The inter-individual variability was smaller for absolute concentrations (internal reference) as compared to that for signal intensity ratios. Significant regional variability in concentration was found for all metabolites, indicating that separate normative values are needed for different brain regions. The values obtained in this study can be used as reference in future studies, provided the same methodology is followed; it is confirmed that despite unsuccessful attempts in the past, smaller coefficients of variation can indeed be obtained through absolute quantification.

## Introduction

In proton magnetic resonance spectroscopy (^1^H-MRS), the signal intensity i s not only proportional to metabolite concentration but is also affected by a large number of variables, including metabolite relaxation rates, pulse sequence parameters and radio-frequency coil sensitivity. As a consequence of the inherent difficulties in obtaining reliable absolute concentrations, the majority of *in-vivo* MR spectroscopy studies of the brain report relative measurements, calculated assuming that the concentration of creatine is constant and taking its signal as reference. However, the interpretation of relative data can be ambiguous when the concentration of creatine changes, such as in early development and in brain tumors.[1,2] In addition to removing interpretation ambiguities, the provision of absolute measurements considerably simplifies the comparison of data recorded under different experimental conditions and the comparison with in-vitro biochemical measurements.[3]

Absolute metabolite concentrations may be derived using an internal reference, usually unsuppressed tissue water, or an external reference, generally water or sodium acetate contained in a tube positioned next to the head of the patient. Both methods have important limitations: the former can suffer from bias due to altered tissue water content, for example caused by edema, and the latter is associated with large inter-subject variability due to coil sensitivity inhomogeneities. Other methods based on replacement phantoms and coil-loading measurements also exist but are less frequently used.[4–8]

In order to obtain absolute concentrations, it is also necessary to determine the relaxation rates of the metabolites of interest in order to correct for transverse and longitudinal magnetization effects. Direct measurement with relaxometry is the most accurate option but is often impractical due to scan time limitations. As a consequence, metabolite relaxation rates are frequently assumed to be constant, and standard values from literature are used; this can be a source of error due to pathology-related changes and regional differences.[9–12]

Despite these difficulties, in recent years quantitative ^1^H-MRS of the brain has gained increased acceptance in the clinical domain and has been applied to the study of aging, senile dementia, epilepsy, multiple sclerosis and neuropsychiatric disorders, among others; it provides a tool for quantitative monitoring of disease progression and treatment response and can support differential diagnosis between conditions characterized by similar imaging findings.[13–17]

There remains, however, a relative paucity of normative studies covering multiple cortical and subcortical brain regions, as well as a large inter-study variability in reported metabolite concentrations (for the cerebral hemispheres, in the range 4-8 mM for inositol compounds, 1-5 mM for choline units, 6-14 mM for total creatine, and 10-25 mM for N-acetyl moieties) and inter-subject variation coefficients (between about 10% and more than 50%).[3,5–8] It has been shown that differences in data processing are a dominant source of inter-study variability; for example, LCModel and AMARES, two widely used commercially available spectroscopy toolkits, may perform differently in terms of sensitivity to noise, linewidth and baseline.[18,19]

Recently, an open-source tool for quantitation of short echo-time spectra, known as AQSES, has become available. In AQSES, the quantitation problem is formulated as a separable nonlinear least-squares fitting problem, and facilities for baseline fitting, removal of residual water and filtering are provided.[20]

In order to corroborate and extend the existing literature, in this study we determined the normal absolute and relative metabolite concentrations in an extended set of brain regions: frontal and parietal white matter, medial temporal lobe, thalamus, pons and cerebellum. Importantly, another aim was to compare the inter-subject variability of absolute concentrations calculated using the internal and external references with that of relative concentrations. This was done with the purpose of determining whether performing absolute quantification is truly justified, in spite of the need to apply multiple corrections involving parameters which inject measurement uncertainty.

## Materials and Methods

### Participants

Twenty-eight right-handed healthy volunteers, 14 female and 14 male, aged 41.2±11.9 years (mean±SD), were enrolled. All subjects, unpaid, were informed about the purpose and clinical relevance of the study, and written informed consent was obtained from them according to institutionally approved procedures and regulations. None of them had a positive anamnesis for neurological or psychiatric disorders, and all had normal magnetic resonance imaging findings (see below).

### Data acquisition

Data acquisition was performed with a Siemens Magnetom Avanto 1.5 T scanner (Siemens AG, Erlangen, Germany) equipped with a transceiver birdcage head coil. For the purpose of positioning the voxels for spectroscopy and to exclude pathology, all subjects were imaged with axial, coronal and sagittal T_2_-weighted turbo spin-echo sequences, with TR=6270 ms, TE=113 ms, field-of-view 260×190 mm, 25 slices, slice thickness 4 mm and no gap. A coronal fluid-attenuated inversion-recovery (FLAIR) sequence was also used, with TR=8700 ms, TE=121 ms, TI=2400 ms, field-of-view 240×190 mm, 20 slices, slice thickness 4 mm and no gap. High-resolution T_1_-weighted volumetric images were acquired by means of an inversion-recovery rapid gradient-echo sequence with TR=1160 ms, TE=4.1 ms, TI=600 ms, field-of-view 270×203 mm, 192 slices, slice thickness 1.1 mm and no gap.

Spectroscopy of the ^1^H nucleus was performed using a single-voxel (SVS) point-resolved spectroscopy (PRESS) sequence, with TR=1500 ms, TE=30 ms and voxel size 20×20×20 mm [with the exception of the medial temporal lobe region (see below), for which it was 40×10×20 mm]; for each voxel, 1024 data points were acquired with a dwell time of 1 ms and 128 averages. Metabolite spectra were obtained suppressing the water signal by means of a chemical shift-s elective saturation (CHESS) pulse with bandwidth 35 Hz. For each spectroscopy voxel, the signal of unsuppressed water was also acquired, with 16 averages. First- (X, Y and Z), as well as second-order (XY, ZX, XY, Z^2^ and X^2^ -Y^2^ ), shimming was performed automatically by means of a field map-based algorithm and then refined manually by an experienced operator; the width at half height of the water peak was generally below 10 Hz (7±2.2 Hz). A 50-mL polystyrene tube containing distilled water, positioned in contact with the head next to the left earlobe, served as external reference; the corresponding water signal was acquired using a 10×10×10-mm voxel.

The static field homogeneity, coil sensitivity maps, tuning and signal-to-noise ratio, radio-frequency chain linearity and gradient performance were routinely checked using the standard software and procedures provided by the scanner manufacturer, with the prescribed periodicity.

**Figure 1:**
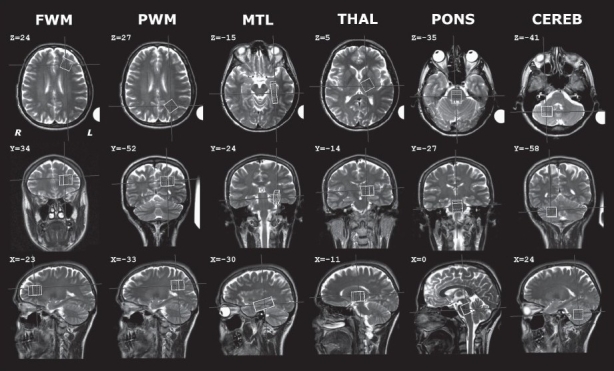
Positioning of the voxels used for acquisition of the spectra, shown on the transverse, coronal and sagittal planes for a randomly chosen subject. The stereotactic coordinates of the voxel centers are given in MNI format. The polystyrene tube positioned next to the left earlobe is visible

Six spectroscopy voxels were positioned in the following regions, shown in Figure 1, together with the corresponding stereotactic coordinates given in Montreal Neurological Institute (MNI) format: frontal white matter (FWM), parietal white matter (PWM), medial temporal lobe (MTL), thalamus (THAL), pons (PONS) and cerebellar hemisphere (CEREB). Notably, as a consequence of choosing equal volume of 8 mL for all voxels, it was not possible to position the voxel entirely within the pons and thalamus; as visible in Figure 1, a contribution of surrounding structures was also present. The Niewenhuys atlas[21] served as anatomical reference. The spectroscopy voxels were normally positioned in the left hemisphere; however, for 9 subjects the cerebellar voxel was placed in the right hemisphere because magnetic susceptibility effects resulted in a water peak width > 10 Hz in the left hemisphere.[22] For 19 subjects, spectra from the right medial temporal lobe were also available; they were not acquired for all subjects due to variable scan-time limitations.

### Preprocessing and quantitation of *^1^H* spectra

**Figure 2:**
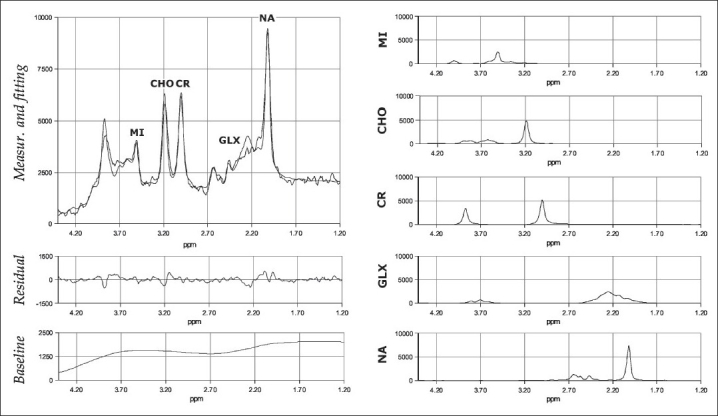
Measured and fitted spectra from a randomly chosen acquisition. The contribution of each component (MI, CHO, CR, GLX and NA), the baseline and the resulting residuals are visible

Spectra were processed by means of the freely available AQSES software (Katholieke Universiteit Leuven, Leuven, Belgium) running under MatLab 7 (The MathWorks Inc., Natick, MA, USA) on a Sun Ultra 80 workstation (Sun Microsystems Inc., Palo Alto, CA, USA).[20] The signal was zero-padded to 4096 points, apodized with a Gaussian function and Fourier-transformed. Zero-order rephasing and frequency correction were performed maximizing the symmetry of the metabolite peaks and centering the N-acetyl moieties peak (see below) on 2.02 ppm. The residual water signal (4.7 ppm) was removed in the range 4.3-5.1 ppm by means of the Hankel-Lanczos singular-value decomposition (HLSVDpro).[20] Remaining baseline fluctuations were removed by fitting with a fifth-degree polynomial. As shown in Figure 2, the metabolite spectrum was fit in the range 1.2-4.3 ppm with the linear superposition of five components, corresponding to inositol compounds (MI), choline units (CHO), creatine and phosphocreatine (CR), glutamate and glutamine (GLX), and N-acetyl (NA) moieties. The intensity of the metabolite signals was determined integrating the following resonance peaks: 3.50 ppm for MI, 3.20 ppm for CHO, 3.00 ppm for CR, 2.70-1.70 (range) ppm for GLX, and 2.02 ppm for NA.[3,5–6] The basis set, provided with the software, was derived from phantom measurements.[20]

Volumetric images were normalized to the MNI space and segmented using the SPM5 program (Wellcome Neuroimaging Department, London, UK), and the relative content of white matter (WM), gray matter (GM) and cerebrospinal fluid (CSF) in each spectroscopy voxel was determined.

Absolute concentrations, expressed in millimolarities (mmol/L, mM), were calculated for MI, CHO, CR and NA (not for GLX, due to the inability to separate glutamate and glutamine at a field strength of 1.5 T), using the intensity of unsuppressed water signal in the spectroscopy voxel (internal reference) or in the polystyrene tube (external reference) as reference. Metabolite concentrations *C*_met_ were determined using

(1), $$C_{\mathrm{met}}\;=\;\frac{k_{\mathrm{met}}\;S_{\mathrm{met}}\;V_{H_{2}O}\;n_{H_{2}O}\;c_{H_{2}O}}{k_{H_{2}O}(1\;-\;f_{\mathrm{CSF}})S_{H_{2}O}V_{\mathrm{met}}n_{\mathrm{met}}}$$

where *k*_met_ and *k*_H_2_O_ are correction factors for T_1_ recovery and T_2_ relaxation of metabolite and water (see below), *S*_met_ and *S*_H_2_O_ are the signal intensities, n_met_ and n_H_2_O_ are the numbers of protons (4 for MI, 9 for CHO, 3 for CR and NA and 2 for water), *f*_CSF_ is the fraction of cerebrospinal fluid in the spectroscopy voxel, V_met_ is the volume of the spectroscopy voxel (8 mL), V_H_2_O_ is the volume of the voxel used for water signal acquisition (8 mL for internal reference, 1 mL for external reference), and *C*_H_2_O_ is the reference water concentration.

The correction factor for metabolite, *k*_met_, was determined using

(2), $$k_{\mathrm{met}}\;=\;\left(\frac{f_{\mathrm{GM}}}{(1-e^{-\mathrm{TR}/T_{1,\mathrm{met},\mathrm{GM}}})e^{-\mathrm{TE}/T_{2,\mathrm{met},\mathrm{GM}}}}\;+\;\frac{f_{\mathrm{WM}}}{(1-e^{-\mathrm{TR}/T_{1,\mathrm{met},\mathrm{WM}}})e^{-\mathrm{TE}/T_{2,\mathrm{met},\mathrm{WM}}}}\right)\frac{1}{1-f_{\mathrm{CSF}}}$$

where *f*_GM_ and *f*_WM_ are the fractions of gray and white matter in the spectroscopy voxel, and, T_1, met, GM_, T_2, met, GM_, T_1, met, WM_, and T_2, met, WM_ are the relaxation times for the metabolite, obtained from[5] and provided in Table 1. Notably, the factor 1/(1-*f*_CSF_) appears in both eq. 1 and eq. 2; whereas in eq. 1 it accounts for the fact that metabolite signal is not received from CSF, in eq. 2 it accounts for the fact that the weighing factors for the relaxation terms, *f*_GM_ and *f*_WM_, may not sum to 1.

**Table 1:** Assumptions about relaxation times and water concentrations made for absolute quantifi cation of metabolite concentrations

	*^T^1,GM*	*^T^2,GM*	*^c^GM*	*^T^1,WM*	*^T^2,WM*	*^c^WM*	*^T^1,CSF*	*^T^2,CSF*	*^c^CSF*
MI	1130 ms	279 ms	-	1200 ms	197 ms	-	-	-	-
CHO	1390 ms	401 ms	-	1440 ms	325 ms	-	-	-	-
CR	1320 ms	204 ms	-	1300 ms	209 ms	-	-	-	-
NA	1330 ms	399 ms	-	1380 ms	483 ms	-	-	-	-
H_2_O	670 ms	76 ms	52.3 mM	510 ms	67 ms	45.8 mM	2400 ms	160 ms	55.5 mM

For quantitation with the internal reference, the correction factor for water, *k*_H_2_O_, was determined using

(3), $$k_{H_{2}O}\;=\frac{f_{\mathrm{GM}}}{(1-e^{-\mathrm{TR}/T_{1,H_{2}O,\mathrm{GM}}})e^{-\mathrm{TE}/T_{2,H_{2}O,\mathrm{GM}}}}+\frac{f_{\mathrm{WM}}}{(1-e^{-\mathrm{TR}/T_{1,H_{2}O,\mathrm{WM}}})e^{-\mathrm{TE}/T_{2,H_{2}O,\mathrm{WM}}}}+\frac{f_{\mathrm{CSF}}}{(1-e^{-\mathrm{TR}/T_{1,H_{2}O,\mathrm{CSF}}})e^{-\mathrm{TE}/T_{2,H_{2}O,\mathrm{CSF}}}}$$

where T_1, H_2_O, GM_, T_2, H_2_O, GM_, T_1, H_2_O, WM_, T_2, H_2_O, WM_, T_1, H_2_O, CSF_, and T_2, H_2_O, CSF_ are the relaxation times for water, provided in Table 1. For quantitation with the external reference, in the calculation of *k*_H_2_O_ the relaxation times of cerebrospinal fluid were used, with *f*_GM_=*f*_WM_=0 and *f*_CSF_=1, and *k*_H_2_O_ was multiplied by a factor of 0.80, determined from coil sensitivity maps acquired during quality assessment checks, to account for the difference in coil sensitivity between the two positions: center of the head and coil and average position of the polystyrene tube.

For quantitation with the internal reference, the concentration of water in the voxel, *C*_H_2_O_, was determined using

(4), $$c_{H_{2}O}\;=\;f_{\mathrm{GM}}c_{H_{2}O,\mathrm{GM}}\;+\;f_{\mathrm{WM}}c_{H_{2}O,\mathrm{WM}}\;+\;f_{\mathrm{CSF}}c_{H_{2}O,\mathrm{CSF}}$$

where *C*_H_2_O, GM_, *C*_H_2_O, WM_, and *C*_H_2_O, CSF_ are the concentrations of water in gray matter, white matter and cerebrospinal fluid, provided in Table 1.[8] For quantitation with the external reference, in the calculation of *C*_H_2_O_ we set

$$f_{\mathrm{GM}}\;=\;f_{\mathrm{WM}}\;=\;0\;\mathrm{and}\;f_{\mathrm{CSF}}\;=\;1$$

In addition to the absolute concentrations, the signal intensity ratios MI/CR, CHO/CR, GLX/CR and NA/CR were also obtained. As these were not normally distributed, the ratios were logarithm-transformed.

### Statistical analysis

For each parameter of interest, mean and standard deviation w ere computed. To ensure that there were no significant deviations from Gaussian distribution, Kolmogorov-Smirnov normality tests were performed.

Inter-subject coefficients of variation (CVs) were calculated for the absolute concentrations and for the signal intensity ratios using the formula CV = SD / mean, and averaged for each metabolite across the six regions. To test for systematic differences between the metabolite concentrations calculated with the internal and external references, paired *t*-tests were performed.

For those subjects for whom spectra were available for both the left and the right MTL, lateralization indices (LIs) for the concentrations of MI, CHO, CR and NA, determined using the internal reference, were computed with the formula

(5), $$\mathrm{LI}\;=\;2(c_{L}\;-\;c_{R})/(c_{L}\;+\;c_{R})$$

where *C*_L_ and *C*_R_ refer to the concentrations in the left and right voxels; for each metabolite, the statistical significance of lateralization was evaluated by means of paired *t*-tests.

To test for differences in metabolite concentrations (internal reference) and signal intensity ratios among brain regions, analysis of variance (ANOVA) was performed, followed by Bonferroni-corrected post-hoc *t*-tests.

## Results

**Figure 3:**
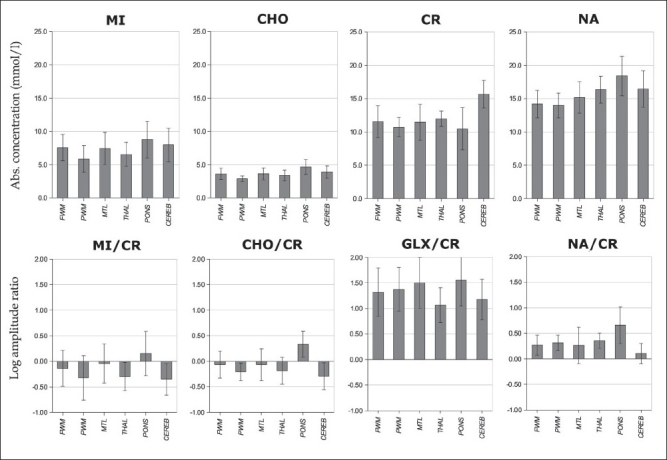
Bar charts of the absolute concentrations (internal reference) and logarithm-transformed signal amplitude ratios. The error bars correspond to 1 SD

The absolute metabolite concentrations and intensity ratios are given in Table 2, and the corresponding bar charts are shown in Figure 3. All concentrations and logarithm-transformed intensity ratios were normally distributed. Across the spectra of the participants, the following average metabolite signal-to-noise ratios (SNRs) were obtained: 32.1±5.1 for CEREB, 28.7±6.6 for FWM, 26.8±6.7 for MTL, 27.3±7.5 for PONS, 28.6±6.9 for PWM and 26.2±4.8 for THAL.

**Table 2:** Absolute concentrations (internal and external references) and logarithm-transformed intensity ratios of the metabolites, and relative voxel contents

	*FWM*	*PWM*	*MTL*	*THAL*	*PONS*	*CEREB*
Internal reference (absolute concentrations in mM):						
MI	7.6±2.0	5.8±2.0	7.9±3.0	6.6±1.8	8.8±2.8	8.0±2.5
CHO	3.6±0.8	2.9±0.4	3.6±1.1	3.4±0.8	4.7±1.1	3.9±0.9
CR	11.5±2.4	10.7±1.5	12.0±4.0	12.0±1.1	10.5±3.2	15.7±2.1
NA	14.2±2.0	14.0±1.8	14.1±2.5	16.3±2.0	18.4±3.0	16.4±2.8
Externalreference (absolute concentrations in mM):						
MI	7.5±3.3	5.9±2.0	8.1±4.1	7.1±4.9	8.2±3.2	8.4±5.5
CHO	3.8±1.8	3.0±0.8	3.8±2.0	3.5±1.6	4.6±1.9	4.1±2.1
CR	11.9±4.9	11.0±3.2	12.4±5.9	12.6±5.2	10.3±4.3	16.6±7.1
NA	14.8±6.4	14.3±3.7	14.6±6.5	17.4±8.6	18.2±6.8	17.4±7.5
Logarithm-transformed intensity ratios:						
ln(MI/CR)	-0.14±0.35	-0.32±0.43	-0.04±0.38	-0.30±0.28	0.15±0.43	-0.35±0.31
ln(CHO/CR)	-0.07±0.27	-0.21±0.17	-0.07±0.31	-0.19±0.26	0.33±0.26	-0.29±0.27
ln(GLX/CR)	1.32±0.47	1.38±0.43	1.50±0.49	1.07±0.34	1.56±0.51	1.17±0.40
ln(NA/CR)	0.27±0.20	0.32±0.15	0.26±0.36	0.36±0.15	0.66±0.36	0.10±0.20
Voxel content:						
% WM	80.4±8.1	81.9±7.1	34.4±10.5	14.0±4.7	48.8±19.1	39.0±15.7
% GM	18.9±7.3	16.2±7.3	61.7±10.7	85.5±5.5	46.4±18.7	60.1±15.1
% CSF	0.7±1.3	1.8±1.9	3.9±3.1	0.5±1.3	4.7±3.4	0.8±1.8

(values are expressed as mean±SD)

There were no significant differences between the average metabolite concentrations calculated with the internal and external references. The average CVs for concentrations determined with the internal reference were 32% for MI, 23% for CHO, 20% for CR and 15% for NA. In comparison, the CVs obtained with the external reference were larger: 50% for MI, 44% for CHO, 41% for CR and 41% for NA. The CVs for the corresponding signal intensity ratios were intermediate: 41% for MI/CR, 27% for CHO/CR, 28% for NA/CR and 48% for GLX/CR.

The lateralization indices for the MTL were 0.01±0.18 for MI, 0.05±0.18 for CHO, - 0 .01±0.18 for CR and 0.01±0.10 for NA; there was no significant lateralization.

The difference in metabolite concentrations (internal reference) among regions was largest for NA (*F*_5, 27_=16.1, *P*< .001), intermediate for CR (*F*_5, 27_=13.5, *P*< .001) and CHO (*F*_5, 27_=11.0, *P*< .001), and smallest for MI (*F*_5, 27_=5.0, *P*< .001). The concentration of MI ranged between 5.8 mM (PWM) and 8.8 mM (PONS), that of CHO ranged between 2.9 mM (PWM) and 4.7 mM (PONS), that of CR ranged between 10.5 mM (PONS) and 15.7 mM (CEREB), and that of NA ranged between 14.0 mM (PWM) and 18.4 mM (PONS).

The difference in transformed intensity ratios among regions was largest for CHO/CR (*F*_5, 27_=19.3, *P*< .001), intermediate for NA/CR (*F*_5, 27_=13.9, *P*< .001) and MI/CR (*F*_5, 27_=7.4, *P*< .001), and smallest for GLX/CR (*F*_5, 27_=5.2, *P*< .001). The transformed intensity ratio ln(MI/CR) ranged between –0 .35 (CEREB) and 0.15 (PONS), ln(CHO/CR) ranged between –0.29 (CEREB) and 0.33 (PONS), ln(GLX/CR) ranged between 1.07 (THAL) and 1.56 (PONS), and ln(NA/CR) ranged between 0.10 (CEREB) and 0.66 (PONS).

The results of post-hoc comparisons between regions are given in Table 3.

**Table 3:** Results of post-hoc comparisons for absolute concentrations (internal reference) and signal intensity ratios (↑ (higher) and ↓ (lower) indicate statistical significance at *P*< .05; ↑↑ and ↓↓ indicate statistical significance at *P*< .01)

	*FWM*	*PWM*	*MTL*	*THAL*	*PONS*
*vs*. PWM		-	-	-	-
*vs*. MTL		MI↓↓, CHO↓,	-	-	-
		MI/CR↓			
*vs*. THAL	NA↓,	NA↓↓	NA↓↓, GLX/CR↑↑	-	-
*vs*. PONS	CHO↓↓, NA↓↓, CHO/CR↓↓, NA/CR↓↓	MI↓↓, CHO↓↓, NA↓↓, MI/CR↓↓, CHO/CR↓↓, NA/CR↓↓	CHO↓↓, NA↓↓, CHO/CR↓↓, NA/CR↓↓	MI↓, CHO↓↓, NA↓, MI/CR↓↓, CHO/ CR↓↓, NA/CR↓↓, GLX/CR↓↓	
*vs*. CEREB	CR↓↓, NA↓↓, CHO/CR↑	MI↓, CHO↓↓, CR↓↓, NA↓↓, NA/CR↑	CR↓↓, NA↓↓, MI/ CR↑↑, CHO/CR↑↑, GLX/CR↑	CR↓↓, NA/CR↑↑	CHO↑↑, CR↓↓, NA↑, MI/CR↑↑, CHO/ CR↑↑, NA/CR↑↑, GLX/CR↑

## Discussion

While there were no systematic differences between the metabolite concentrations estimated with the internal and external references, the external reference was associated with considerably larger inter-individual variability, reflected in coefficients of variation about twice as large. This finding is in line with the results obtained at the majority of centers that participated in a previous multi-centric study.[7] Imperfect repeatability in the positioning of the reference tube is the main source of random error, and its effect is amplified by changes in the coil sensitivity profile between subjects, caused by varying shape and size of the head. It therefore appears generally preferable to use the internal reference unless alterations in tissue water content are expected; more successful results with exernal standards have, however, been obtained by some groups.[6,7,23]

The inter-subject coefficients of variation were larger (by about 40%) for the signal intensity ratios than for the corresponding absolute concentrations estimated with the internal reference. This likely results from the combination of two factors: 1) the actual inter-individual variability in concentration is lower for water (as confirmed by proton density-mapping studies) than for creatine, and 2) the water peak is much larger than the creatine peak and therefore associated with a smaller relative measurement error.[24]

One common criticism to absolute quantification is that it involves the use of multiple correction parameters, namely, *C*_H_2_O_, *f*_GM_, *f*_WM_, *f*_CSF_, T_1, met, GM_ T_2, met, GM_, T_1, met, WM_, T_2, met, WM_ which carry uncertainties that are difficult to estimate and which get propagated in the final measurement result. Although this is an important shortcoming, our findings on the inter-subject coefficients of variation (representing random error) demonstrate that performing absolute quantification is nevertheless motivated, potentially leading to increased sensitivity to pathological change when compared to metabolite ratios.

Several studies reported metabolite concentrations in the cerebral hemispheres: the average concentrations obtained in the present work (7 mM for MI, 3 mM for CHO, 11 mM for CR and 14 mM for NA) and the corresponding inter-individual CVs are within the spread of values found in literature (4-8 mM for MI, 1-5 mM for CHO, 6-14 mM for CR and 10-25 mM for NA).[3,5–8]

The NA peak is frequently assumed to correspond to N-acetyl aspartate, a compound of interest as a putative neuronal marker. *Ex-vivo* studies of the human and rat brain have reported concentrations in the range 5-8 mM, significantly below the estimates obtained in this study and in most other *in-vivo* MRS studies.[25,26] It is hypothesized that this discrepancy arises because of unresolved compounds contributing to the 2.02 ppm NA resonance observed at 1.5 T, and possibly due to N-acetyl aspartate loss during *ex-vivo* sample preparation.[3,5] The CR peak at 3.00 ppm corresponds to the superposition of creatine and phosphocreatine, which have well-known roles in cell metabolism, in the concentration ratio determined by phosphokinase equilibrium.[5] *Ex-vivo* studies of the human and canine brain have reported concentrations in the range 9-12 mM, in line with the results of this study and other *in-vivo* MRS studies.[3,5,27–28] The CHO peak at 3.20 ppm is the most complex, receiving contributions from a range of choline-containing compounds, including phosphocholine, glycerophosphocholine, free choline, acetylcholine, phosphatidylcholine and choline-plasmalogen; its intensity is frequently taken as an empirical marker of the density and turnover of cell membranes.[3,5] In vitro, the concentration of phosphocholine, glycerophosphocholine plus free choline was determined to be in the range 1-2 mM, in line only with some *in-vivo* studies but below the estimates of others (including the present one); the determinants of this discrepancy remain unclear.[3,5–6,8,29] The MI peak at 3.50 ppm corresponds to a range of compounds, including phosphatidylinositol, inositol polyphosphatide, inositol monophosphate, myo-inositol and, to a smaller extent, glycine; as inositol is elevated within astrocytes, the intensity of the peak is often taken as an empirical marker of glial density and proliferation. Studies on human brain samples have reported concentrations in the range 5-7 mM, in line with the results of this study and other *in-vivo* MRS studies.[6,8,25,30] The GLX complex in the range 2.70-1.70 ppm includes multiple overlapping resonances from glutamate and its precursor glutamine; as these cannot be resolved at 1.5 T, quantitation was not attempted.[6]

In agreement with the existing literature, significant regional differences were found for all metabolites of interest.[6,8,22] These were largest for NA, intermediate for CR and CHO, and smallest for MI. Given that N-acetyl aspartate is present in the soma of neurons, in dendrites and in axons, its regional variability is likely related to differences in neural architecture, population and density; a simple linear relationship with the density of neurons is, however, unlikely given that it also reflects reversible metabolic changes.[31] Total creatine and choline are less specific as they include contributions from both neurons and glia, and their regional variability is probably related to differences in the density of the cellular matrix; and in the case of choline, also in the level of myelination.[32] The variability in the concentration of inositol compounds could be more specifically related to the glial population, but it is likely also influenced by regional metabolic differences.[6]

The concentration of N-acetyl aspartate was higher in the thalamus, pons and cerebellum than in the cerebral hemispheric regions; this effect could be related to higher density of neural soma, axons and dendritic trees in these regions, and is found for the thalamus also in[8] but not in.[6] The concentration of choline units was markedly higher in the pons than in other regions; this effect could be related to the high myelination of the dense rostro-caudal pathways in this region[21] and is also found in[6] and[8]. In agreement with the same studies, the concentration of choline units was lowest in the parietal lobe; the same trend was observed for inositol, and the interpretation is unclear. The concentration of creatine was markedly higher in the cerebellum than in other regions; this effect could be related to the very high density of neural cells in the cerebellum[21] and is also found in[6] and[22]. The presence of significant regional differences signals the need to have separate reference values for each region of interest, especially for subcortical structures.

The concentration of metabolites in the hippocampus was found to be symmetrical, in line with[33] significant asymmetries emerge in epilepsy, even in absence of frequent seizures.[15]

The present study has three main strengths. First, in contrast with several previous ones which considered specific regions only, metabolite concentrations were measured, for all subjects, in an extended set of cortical and subcortical brain regions. Second, high-resolution segmentation was performed to correct for cerebrospinal fluid partial voluming and to report the white/ gray matter contents of each voxel. Third, a freely available fitting program was used, enabling centers that do not have access to commercial spectroscopy software to reproduce our findings.

There are, however, also several important limitations that need to be taken into consideration. First, the metabolite relaxation rates were not measured, due to scan-time limitations related to the large number of voxels under study, and the values from a previous study were used. While this is a potential source of error, it should be noted that the inter-study variability in relaxation times is considerably smaller (CV< 20%) in comparison with that in concentrations.[9] There are known differences in relaxivities among brain regions, which are less marked at 1.5 T than at higher field strengths; these were indirectly taken into account, determining “effective” T_1_ and T_2_ for each metabolite on the basis of the content of each voxel and of the average T_1_ and T_2_ values for white and gray matter.[5,9–11] Although this approach cannot reveal pathology-induced changes in the metabolite T_2_ s as found, for example, in stroke, the potential confounding effect was minimized because the T_2_ -weighting was very weak, given that the MRS echo time (30 ms) was considerably shorter than the metabolite T_2_s (200-500 ms).[9,12] Second, the uncertainty associated with each correction parameter (*C*_H_2_O_, *f*_GM_, *f*_WM_, *f*_CSF_, T_1, met, GM_, T_2, met, GM_, T_1, met, WM_, T_2, met, WM_) remains unknown, and the finding of reduced inter-subject coefficients of variation does not exclude the presence of significant systematic error, even though there were no systematic differences between internal and external references. This is a general limitation of the technique at the present stage, which needs addressing through dedicated relaxometry and segmentation studies. Our findings of reduced inter-subject variability motivate such studies. Third, with the exception of the medial temporal lobe, voxels were positioned in one hemisphere only, preventing the determination of potential metabolic asymmetries related, for example, to the known difference in axonal density in some regions between the dominant and the non-dominant hemispheres; this limitation is in common with many other similar studies.[3,5,7–8] Fourth, the number of subjects and the age range were too small to investigate age-related changes. The age range under consideration is, however, relevant to a wide range of pathologies, and previous studies indicate that the age-related changes in metabolite concentrations are negligible until the seventh decade.[32] Another limitation is that the study was conducted at 1.5 T, while the use of a 3-T scanner would have provided better SNR and peak separation, reducing quantitation uncertainty; however, in the majority of centers, clinical spectroscopy is still routinely practiced at 1.5 T.[34] Due to the inability to resolve glutamine and glutamate resonances at 1.5 T, the data on the GLX/CR ratio should be interpreted with caution. Finally, the voxel size (8 mL) was comparatively large, especially considering the pons and thalamus regions. Although we have corrected for partial voluming with fluid-containing spaces, inclusion of gray and white matter from surrounding areas affected the anatomical specificity of the measurements. Reducing voxel volume, e.g., to 4 mL, would increase anatomical specificity, as well as limit susceptibility effects. In this study, the choice of 8 mL was made in order to maximize SNR, and all voxels had equal volume to avoid introducing SNR differences across the regional spectra. It has been previously shown that reducing the SNR increases random error and may also result in systematic concentration biases.[19,34] While comprehensive evaluations of the effects of SNR are available for LCModel and AMARES, at present these remain lacking for AQSES.[19] Additional work is therefore necessary both to determine how SNR changes affect the accuracy of concentrations calculated using the proposed method, and to derive further reference values using smaller voxels.

More generally, a limitation of the present study, which is in common with all other similar normative studies,[3,5–8] is that the reference values are valid only so long as the same methodology and conditions are applied. Replication of the quantification procedure employed in the present study appears unproblematic as a full description has been g iven and the AQSES software is freely available. The exact position of the voxels in normalized space was provided [Figure 1], and the acquisition parameters are standard for clinical MRS at 1.5 T. Nevertheless, there are a number of potential sources of error, such as differences in signal-to-noise ratio, shimming quality and pulse sequence implementation, as a consequence of which our findings cannot immediately be used as normal reference even in absence of explicit methodological differences; rather, they should initially be compared with locally acquired control values and included as reference only after confirming the absence of statistically significant differences. For situations in which the same quantification methodology cannot be followed, for example when a different value of field strength is used, the present values cannot be considered and the relevance of the present work is purely methodological.

In conclusion, we have reported the normal *in-vivo* concentrations of inositol compounds, choline units, total creatine and N-acetyl moieties in frontal and parietal regions, medial temporal lobe, thalamus, pons and cerebellum. These values can be used as reference for future studies provided the same methodology, based on open-source software, is followed. The inter-individual coefficients of variation were largest for quantitation based on an external reference, discouraging its general use; there was, however, no systematic bias with respect to quantitation based on tissue water. The inter-individual coefficients of variation were larger for metabolite ratios than for absolute concentrations, confirming the potential usefulness of quantitative MRS in spite of the complexity of the correction process. Significant differences in metabolite concentrations were found among brain structures. While their exact determinants cannot be determined by *in-vivo* studies such as the present one, they are hypothesized to be related to regional variability in neural and glial population, and in myelination. Separate reference values are needed for different brain regions.
